# Comparison and Impact of Four Different Methodologies for Identification of Ambulatory Care Sensitive Conditions

**DOI:** 10.3390/ijerph17218121

**Published:** 2020-11-03

**Authors:** Andreia Pinto, João Vasco Santos, Júlio Souza, João Viana, Cristina Costa Santos, Mariana Lobo, Alberto Freitas

**Affiliations:** 1 Department of Community Medicine, Information and Health Decision Sciences (MEDCIDS), Faculty of Medicine, University of Porto, 4200-450 Porto, Portugal; jvasco.santos@gmail.com (J.V.S.); juliobsouza@med.up.pt (J.S.); joao.a.viana@gmail.com (J.V.); csantos@med.up.pt (C.C.S.); nanalobo@gmail.com (M.L.); alberto@med.up.pt (A.F.); 2 CINTESIS–Center for Health Technology and Services Research, 4200-450 Porto, Portugal; 3 Public Health Unit, ACES Grande Porto VIII–Espinho/Gaia, 4500-330 Porto, Portugal

**Keywords:** ambulatory care sensitive conditions, hospitalizations, primary health care, reproducibility of results, Portugal

## Abstract

Ambulatory care sensitive conditions (ACSCs) are conditions for which hospitalizations are thought to be avoidable if effective and accessible primary health care is available. However, to define which conditions are considered ACSCs, there is a considerable number of different lists. Our aim was to compare the impact of using different ACSC lists considering mainland Portugal hospitalizations. A retrospective study with inpatient data from Portuguese public hospital discharges between 2011 and 2015 was conducted. Four ACSC list sources were considered: Agency for Healthcare Research and Quality (AHRQ), Canadian Institute for Health Information (CIHI), the Victorian Ambulatory Care Sensitive Conditions study, and Sarmento et al. Age–sex-adjusted rates of ACSCs were calculated by district (hospitalizations per 100,000 inhabitants). Spearman’s rho, the intraclass correlation coefficient (ICC), the information-based measure of disagreement (IBMD), and Bland and Altman plots were computed. Results showed that by applying the four lists, different age–sex-adjusted rates are obtained. However, the lists that seemed to demonstrate greater agreement and consistency were the list proposed by Sarmento et al. compared to AHRQ and the AHRQ method compared to the Victorian list. It is important to state that we should compare comparable indicators and ACSC lists cannot be used interchangeably.

## 1. Introduction

Ambulatory care sensitive conditions (ACSCs) are conditions that can be typically managed at the first level of health care and for which hospitalizations can be potentially avoidable, in the sense that effective primary health care should provide an early diagnosis and proper management of chronic diseases, reducing the risk of hospitalization [1,2,3].

The determination of hospital admission rates for ACSCs has been increasingly used as an indicator to measure the quality and accessibility of primary health care [4,5,6]. The concept started to gain interest in the early 1990s in the United States (US) [1,7,8] and, over the years, many authors studied the impact of avoidable hospitalizations in different countries [9,10,11,12,13,14,15,16,17]. In some European countries, where access to primary health care (PHC) is universal, the concept of ACSCs is mostly applied to evaluate the quality of primary care, as it happens in Portugal, rather than to evaluate accessibility [10], which is more common, for instance, in the US, where health care access is not universal. Additionally, in other countries with PHC systems similar to the Portuguese system, such as Spain, Italy, France, and Finland, where there is universal health coverage, the gatekeeping function (general practitioners are the entry point to the health system and thus primary care providers coordinate referrals to hospitals and specialists) is practiced and the patient must be registered with a general practitioner; such countries have reported geographical variations in ACSC hospitalizations [16,18,19,20]. Distinguishing this type of variation can be crucial to identify areas that need a deeper focus and possibly further regionally or locally tailored health policies [19].

However, there is a lack of consensus regarding which conditions should be considered as ACSCs. To select the set of ACSCs that is most important to the population under study, different lists have been proposed in the literature, and the choice of the list to be used is considered one of the most important steps to estimate avoidable hospitalization rates [3,21]. ACSCs are usually defined through a consensus method, often using the Delphi method with a panel composed of experts [1,11,22,23,24].

The rate of ACSCs in a specific population is impacted by the list applied in the study, that is, (1) the respective coding of diseases, (2) the version of International Classification of Diseases (ICD), (3) the age group covered, (4) the exclusion criteria, and (5) research setting. Therefore, it varies between contexts [25], causing variability in the results and consequently in their interpretation. At the moment, despite the large number of ACSC lists, many countries do not have a list adapted to their specific context; in general, the choice of the list used to calculate the rates of avoidable hospitalizations is selected according to the choice of the study group.

Previous studies conducted in Portugal and France assessed the impact of using two methodological approaches to identify ACSCs. Kappa statistics for nominal scales were used to assess the agreement in the Portuguese study, and the impact between the different lists was verified graphically in the second study. Both studies verified considerable differences in rates, with poor agreement between the methods [26,27]. It is also relevant to assess whether rates computed based on different lists provide consistent measures (i.e., whether the increase or decrease of hospitalizations is observed in each list to the same extent) and, more importantly, whether they can be exchangeable. Thus, our objective was to compare the impact of ACSC rates by using different ACSC lists.

## 2. Materials and Methods

We conducted a retrospective study in order to assess and compare ACSC hospitalization rates in mainland Portugal using different lists proposed in the literature, for the period between 2011 and 2015, using the district level as the unit of analysis.

Data concerning hospital discharges from all public hospitals were provided by the Central Administration of the Health System (ACSS). Annual estimates of the resident population by district of residence and for all years of the study were extracted from Statistics Portugal [28].

A review of ACSC lists identified in the literature or in well-known organizations was performed to determine which ones could be included in this study. Afterward, we reviewed the method behind each ACSC list, including the diagnosis codes defined as ACSCs, the coding system used (International Classification of Diseases 9th revision (ICD-9) or ICD-10 codes), the inclusion and exclusion criteria (e.g., principal diagnosis or any diagnoses, age group range, exclusion of procedure codes), and the country in which the list is commonly applied or has been validated, as it is generally specific to the country’s health needs and the health system itself. Since in Portugal, the discharges were coded in ICD-9-CM until 2016, we excluded all the ACSC lists defined with ICD-10 codes. Only the list proposed by Sarmento et al. (2020), which is defined in terms of the ICD-10, was converted into ICD-9-CM codes in order to be included in this study, as it was developed for the Portuguese context. The conversion was carried using the General Equivalence Mappings (GEMs) method, implemented in the R package “touch”. Hence, avoidable hospitalizations were identified as ACSCs according to the set of ICD-9-CM codes defined in the lists adopted by the Agency for Healthcare Research and Quality (AHRQ) version 6.0 [13], by the Canadian Institute for Health Information (CIHI) [12], and by the Victorian Ambulatory Care Sensitive Conditions Study (2001-02) [29] and the list of ACSC conditions suggested by Sarmento et al. (2020), which was published very recently [30].

### 2.1. AHRQ List

The AHRQ developed the prevention quality indicators (PQIs), which are computed based on a set of codes of principal or secondary diagnosis that are related to ACSC hospitalizations. This method is well recognized and employed in several studies/countries [31,32,33,34,35,36], although it was firstly developed in the US. We applied in this study the PQI 90 Overall composite, which covers 11 diseases in the adult population (≥18 years old). It excludes obstetric admissions; transfers from other institutions; some procedure codes; and hospitalizations for which patient information regarding sex, year of hospitalization, main diagnosis, or residence is missing.

### 2.2. CIHI List

The CIHI method encompasses only seven conditions and only chronic diseases, which are identified by principal diagnosis for patients younger than 75 years of age. Exclusions are based on some procedure codes, records with death as discharge status, and also newborns and stillbirths.

### 2.3. Victorian List

The Victorian ACSC study includes 19 conditions (acute, chronic, and immunizable diseases) that are identified mostly by principal diagnosis and some diseases through “any diagnosis”. In this method, there is no exclusion for age, only for some procedure codes.

### 2.4. Sarmento et al. List

Recently, Sarmento et al. [30] performed a modified web-based Delphi panel approach to define which conditions are considered ACSCs in the Portuguese adult population context (aged 18 years or older). Experts from the most important Portuguese scientific societies (general practitioners and medicine physicians) selected the conditions by choosing a list of conditions previously identified as ACSCs in the literature that could be considered avoidable in terms of hospital admissions in Portugal. Furthermore, they had to identify other potential conditions that they considered avoidable in terms of hospital admission in Portugal despite not being identified in the previous list. The consensus level was established as 75% after a maximum of three rounds. A core list of ACSCs that met criteria ii to iv proposed by Solberg and Weissman (clarity in the definition and coding of diagnoses; with relevance for public health, that is, a hospitalization rate of least 1/10,000 population; and if the diagnosis is potentially avoidable by timely and effective ambulatory care) [7,8] and an extended list with all the conditions agreed upon by the experts, even if not fulfilling the criteria proposed by Solberg and Weissman, were determined. We applied in this study the core list of Portuguese ACSCs and the conditions were identified by principal diagnosis. Exclusions based on diagnosis or procedure codes have not been defined by Sarmento et al. list. Table 1 includes the diseases that are considered sensitive to primary care and the exclusion criteria applicable in each list.

Of the 9,048.742 admission cases included in the database (2011–2015) provided by ACSS, only 5,682,688 corresponded to episodes of hospitalization (the remaining cases refer to ambulatory episodes and thereby were excluded). Considering all hospitalizations, we only included episodes that are considered statistically valid (*n* = 4,426,227), that is, the length of stay in the hospital was at least 24 h or shorter than 24 h for patients who died, left against medical advice, or were transferred. Additionally, the episodes for which the districts are unknown, as well as admissions related to territories outside mainland Portugal were excluded from the analysis (*n* = 23,072). We also calculated the sum of all avoidable hospitalizations for the diseases that are part of each list (that is, the composite indicator of each list), such as the example of the PQI90 defined by AHRQ.

### 2.5. Statistical Analysis

A descriptive analysis was performed considering the number of hospitalizations each year and the proportion of those that are considered preventable.

For each studied list, all ACSC hospitalization rates were calculated per 100,000 inhabitants (according to the age groups required in each list) by district and year. Age- and sex-standardized ACSC hospitalization rates were calculated through direct standardization using the 2015 Portuguese population data as the reference population to overcome different age distributions across regions in Portugal (there is an older population in the inner cities and a younger population in more populated areas located on the coast), which may contribute to regional variations in the prevalence of some diseases.

We performed the same methods used recently by Santos et al. for assessing the correlation, reliability, and agreement of health expectancies [37]. Spearman’s rho was calculated to assess the correlation of avoidable hospitalization rates between lists. This method provides scores that can vary between −1 and +1, and a value of 0 means that no association exists between the variables [38]. The 95% confidence intervals (CIs) were estimated using a bootstrapping with 1000 resamples. However, the correlation only indicates the direction and degree of association between the variables, so we calculated other measures that allowed us to infer the reliability and agreement between the variables (the avoidable hospitalization rate obtained from the different lists) since two methods can be correlated without a great agreement. Thus, intraclass correlation coefficient (ICC) and information-based measure of disagreement (IBMD) were also calculated in order to study the reliability and disagreement, respectively. ICC was used to measure the reliability of the different lists (continuous variables), and the ICC estimates and 95% CIs were calculated based on a two-way mixed-effects model, consistency definition, and average measurements. The ICC value obtained can range from 0 to 1, with values closer to 1 indicating better reliability [39,40]. IBMD was used to evaluate the disagreement among the rates; coefficients closer to 0 indicate less disagreement among the measures, while coefficients closer to 1 indicate higher disagreement [41]. Bland and Altman [42] plots were also performed to graphically assess the average difference in the rates obtained from the different lists (the agreement), also providing information regarding the variability of rate across districts [43]. Given that rate estimates were obtained per year and district, the average difference in rates drawn for the Bland and Altman plots was determined using mixed-effect models, which can handle the repeated measures of our data.

Analyses were performed using the RStudio Team (Boston, MA, USA), version 1.2.1335, IBM SPSS Statistics, version 26.0 (IBM Corp., Armonk, NY, USA), and Microsoft Excel, version 16.37 (Microsoft Corporation, Redmond, WA, USA).

## 3. Results

After applying the exclusion criteria, it was found that a total of 4,403,155 eligible hospitalizations occurred in the 5 years of the study. In Table 2, the number of episodes of inpatient hospitalizations in Portugal between 2011 and 2015 and the proportion of those considered avoidable are presented, for each methodology and year, according to the inclusion and exclusion criteria of each list.

Analyzing Table 3, it is possible to observe the age–sex-adjusted rates according to the district in 2011 and 2015. Applying AHRQ, the Victorian study, and Sarmento et al. methods, the results showed an overall increase in avoidable hospitalization rates during the 5 years of study (6.1%, 5.5%, and 4.4%, respectively), whereas a decrease of about 4.3% was observed when applying the CIHI list. In Table 3, districts presenting a 10% or more change between the first and last year of analysis are marked with a symbol. Almost all districts showed a rate increase across the 5 years in three out of the four lists; however, only a few displayed an increase or decrease of above 10%. Only CIHI methodology showed a 10% or more decrease in the rates.

Considering the correlations between the methods, according to the calculation of the Spearman’s rho (Table 4), all comparisons represented a positive correlation; that is, as one variable increases, the other variable tends to also increase. The adjusted rates calculated by the AHRQ and Sarmento et al. methods were the ones with the highest correlation (*p* ≥ 0.893), whereas the adjusted rates of the comparison regarding CIHI and Victorian methodologies presented the lowest value, particularly in 2015 (*p* = 0.645). Low levels of association were also observed in other comparisons in which the CIHI method is present. Considering the years studied, 2013 was generally the year with the highest correlation between the studied lists, while 2015 was generally the year in which the correlations were lower.

The IBMD allowed us to make inferences about the disagreement between the different methods (Table 5). Regarding this statistical parameter, we found a greater disparity depending on the pair of lists evaluated. We observed that the pairs of lists that included the CIHI method presented a higher disagreement, as the IBMD value was closer to 1 (IBMD > 0.8). AHRQ and Victorian methods were the ones that showed a smaller disagreement in the 5 years of analysis (IBMD ≤ 0.148), and the values were similar over the years. The list proposed by Sarmento et al. compared to the Victorian methodology showed a greater disagreement (IBMD = 0.407) in relation to the same list compared to the method from AHRQ (IBMD = 0.330).

The results for ICC are presented in Table 5. The comparison of the AHRQ method with Sarmento et al. list showed the highest ICC of all list comparisons (ICC > 0.9 in all years), and the comparison of the AHRQ list with the Victorian method showed the second-highest ICC of all list comparisons. The comparisons that included the CIHI list (CIHI vs. Victorian, CIHI vs. AHRQ, and CIHI vs. Sarmento et al.) presented the lowest values of ICC in all the analyzed years; however, the CI 95% was very broad and had no statistical significance in the two latter comparisons (CIHI vs. AHRQ and CIHI vs. Sarmento et al.).

**Figure 1:**
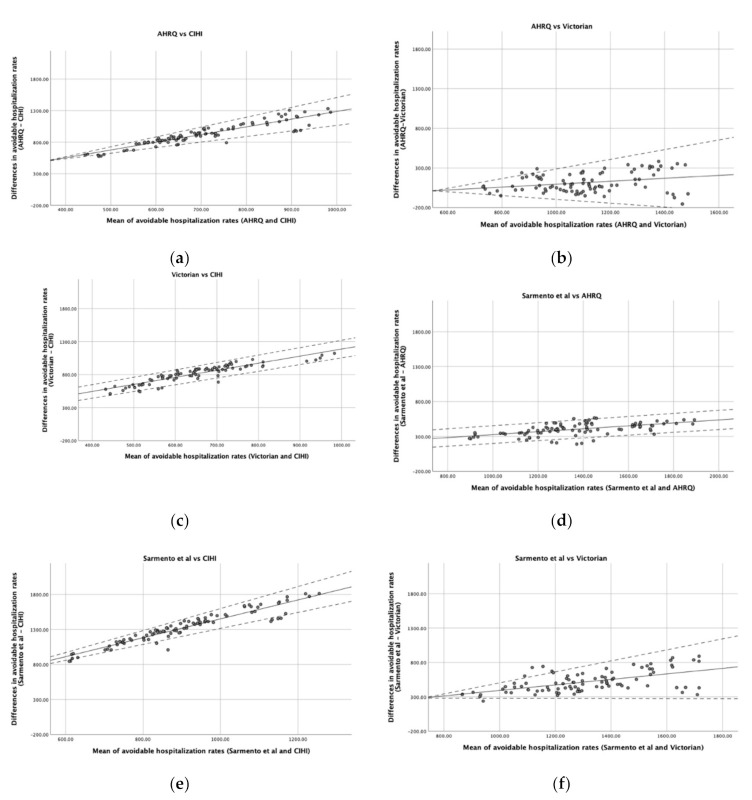
Bland and Altman plots comparing the agreement of different lists. (**a**) AHRQ vs. CIHI; (**b**) AHRQ vs. Victorian; (**c**) Victorian vs. CIHI; (**d**) Sarmento et al. vs. AHRQ; (**e**) Sarmento et al. vs. CIHI; (**f**) Sarmento et al. vs. Victorian. Solid line represents the difference in avoidable hospitalization rates between lists and dashed lines indicate the Bland and Altman limits of agreement.

Figure 1 is composed of six graphs that demonstrate the agreement between the pairs of lists. There is a positive slope in all graphs, indicating a positive average association between the differences in rates and the mean of rates estimated by the two lists. Thus, as the rate of avoidable hospitalizations increases, the greater the overestimation of one list seems to be in relation to the other. Moreover, in some cases (Figure 1a,b,e,f), the variability in the agreement also seems to be dependent on the mean rate of avoidable hospitalizations, being larger in districts in which the mean rate is higher and less noticeable in districts where the rate of avoidable hospitalizations is lower. However, in comparisons such as (c) or (d) of Figure 1, the variability of the differences does not seem to depend on the mean of the rates.

Another parameter that should be taken into account is the range of values regarding the difference between the two lists. When a perfect agreement is obtained, the bias between the two lists is represented by a horizontal line close to 0. Thus, only the comparison (b) of Figure 1 seems to reach agreement in some districts with lower avoidable hospitalization rates, being also the only graph where the 0 appears in the *y*-axis. Nevertheless, for districts with larger rates, the AHRQ rate may either over- or underestimate the Victorian rate by more than 100 hospitalizations/100,000 inhabitants. The other comparisons, in general, presented a range of values regarding the differences between the lists of about hundreds of hospitalizations per 100,000 inhabitants.

## 4. Discussion

In this study, we identified that the AHRQ, the Victorian study, and Sarmento et al. methods showed avoidable hospitalization rates that were more similar across districts in comparison with the CIHI method, where the values found were about one-quarter or less of those found in the other lists. The AHRQ, Victoria study, and Sarmento et al. lists measured avoidable hospitalizations consistently, meaning that a positive trend over the years was identified when applying any of them, whereas a slight decrease in absolute numbers was observed when applying the CIHI list, though the percentage of avoidable hospitalizations remained stable. These results are in agreement with the findings obtained by Sarmento et al. when evaluating the Canadian method (CIHI) and the Spanish list developed by Caminal et al. [9], which was not evaluated in this work. In that study, between the years 2000 and 2012, the authors identified such discrepancies with the application of the CIHI list, as a decrease in the rate of avoidable hospitalizations was verified, in contrast to what was observed when applying the list proposed by Caminal et al. [26].

We obtained a considerably high correlation between all the methods; however, the correlation only allows us to conclude that the variables are associated and does not permit inferences about the agreement between them [44]. For this reason, we calculated the ICC to evaluate the reliability, and we calculated the IBMD and drew Bland and Altman plots to measure the level of agreement between lists. The comparison between the AHRQ list and the Victorian methods showed the almost highest correlation and reliability (ICC) measures and an IBMD value closer to 0. Moreover, the Bland and Altman graph revealed that some districts can have a difference close to 0. These results are quite similar to the ones obtained for the comparison between the AHRQ and Sarmento et al. lists, although the disagreement was relatively high (IBMD = 0.330) compared to the other two lists. Consequently, these two lists seem to be the ones presenting more similar results. Nevertheless, it should be noted that the different methods applied in this study clearly present specificities between them (the diseases considered ACSCs, the associated codes, and the number of cases included and excluded also determine the number of episodes that are included in each list). All of these may explain the differences found in avoidable hospitalization rates. To illustrate, the CIHI method only considers a few chronic conditions as ACSCs. Furthermore, in this method, individuals aged over 75 years old are excluded, and it is known that age is an important factor associated with hospitalization for ACSCs, as chronic conditions are more prevalent in older populations [25], which might explain the lower rates of ACSCs obtained by this list in comparison with the other methodologies. Additionally, it also suggests that the prevalence of chronic conditions associated with avoidable hospitalizations that are included in the CIHI list is stable across the years under study. These reasons may also justify the inverse trend in avoidable hospitalizations compared to the other methods, since all hospitalizations due to acute illness are not counted. This leads us to believe that acute hospitalizations may have a considerable influence on avoidable hospitalization rates.

Moreover, different lists can have distinct purposes; for instance, the lists that cover more chronic diseases focus essentially on the management of primary health care, whereas other lists, such as the Victorian method, can be more comprehensive, including acute and immunizable diseases, and for that reason tend to identify a higher number of hospitalizations. Nevertheless, to counteract these limitations, the construction of an international list would bring advantages regarding comparative purposes; to achieve this goal, the health indicator should cover the same codes related to a specific disease, according to Nedel et al. [45].

Our findings show that avoidable hospitalizations increased in Portugal when considering the lists that included acute and chronic ACSCs and decreased with the list that only includes chronic ACSCs, though rates remained stable. Rosano et al. compared hospitalizations of ACSCs in Italy and Germany and verified that avoidable admissions accounted for 8% and 11% of total admissions, respectively, using the conditions defined by Pirani et al. and the criteria defined by Weissman et al., between the years 2000/2001 and 2008 [46], with the percentages of the hospitalizations due to ACSCs not differing much from those found in our study. Rosano et al. also verified that, in Italy, the chronic conditions decreased by about 23%, in contrast to the acute conditions, which remained stable. On the other hand, the hospitalizations due to ACSCs in Germany increased for both conditions. In an ecological study that compared the potentially avoidable hospitalizations in five European countries (Portugal, Denmark, Spain, England, and Slovenia) through six chronic conditions, the rates in 2009 varied from 93.7 cases per 10,000 inhabitants in Denmark to 34.8 cases per 10,000 inhabitants in Portugal. The authors also concluded that a variation within-country was presented and the rates remained stable throughout the period analyzed, except for in England and Denmark where rates decreased [16]. These findings are similar to ours regarding the application of the CIHI list that similarly only includes chronic conditions, and the rates seemed to be stable across the years.

When comparing our results of ACSC rates to those of other similar international studies in the literature, we must be aware that the comparison should be analyzed with caution or even avoided if the list applied was not the same, also considering the fact that the health context itself differs. Likewise, some authors [10,47] state that the inconsistency of codes and diseases used by different methodologies hampers the comparison between geographical areas, as well as a wider and broader utilization of the indicator. However, there are also advantages of validating a list of ACSCs according to the health context of a given population (or country), since it increases the specificity of the list, which is more suitable for primary health care in that specific health context [26]. A work by the World Health Organization [48] on the evaluation of the provision of health services according to hospitalizations by ACSCs stated that the list developed by Bardsley et al. [49] was the most robust generic non-country-specific list of ACSCs.

Nevertheless, studies that address comparisons between countries considered that the avoidable hospitalization rates seem to be lower in those with stronger primary health care [50]. This reinforces the idea that hospitalizations by ACSCs can be avoided, essentially by good primary health care services. However, in high-income countries, geographic variations are relatively common and can represent variable quality of care and inefficient use of resources [19]. Some studies have already identified several factors that could explain the variation of the avoidable hospitalization rates within countries. Particularly in Portugal, Carneiro et al. showed a statistically significant association between the rates of hospitalizations due to ACSCs and the proportion of the population with family physicians and travel time to the provider. Additionally, despite not being statistically relevant, areas with individuals with lower education and with lower income presented higher ACSC hospitalization rates [51]. Another study carried out in Portugal found that the population density was lower for municipalities with a higher risk of hospitalizations for ACSCs (acute and chronic conditions). Considering acute diseases only, the municipalities that had a higher risk of hospitalization due to ACSCs had a higher mean proportion of elderly people, a higher proportion of people living in rural areas, and a low education level [31]. These factors were also identified in studies conducted in other countries [16,19].

### Limitations and Strengths

We assessed the avoidable hospitalizations in Portugal through four different methodologies, providing different rates of avoidable hospitalizations regarding the list applied. We also adjusted the rates for age and sex to account for age differences in geographic distribution and possibly different disease prevalence being present, which increased the fairness of the comparison between districts. Still, we did not adjust our results to other factors that could yet influence the obtained rates and more final insights regarding the performance of regional providers, such as economic and educational factors and the distance faced by some populations in travel to primary care facilities.

This study was performed with data from mainland Portugal and only included hospitalizations occurring in public hospitals; therefore, other episodes of ACSC hospitalizations may have occurred in private hospitals during the studied period.

The list developed by Sarmento et al. adapted to the Portuguese context was originally defined with ICD-10 codes; however, to convert to ICD-9-CM codes, we first performed an approximation to ICD-10-CM codes using the first three or four ICD-10 characters, and the approximate matches were then mapped to ICD-9-CM using the GEMs method, which in turn may have resulted in some codes being slightly different from the codes defined in the original list.

## 5. Conclusions

Of all the studied lists, those that seemed to demonstrate a greater agreement were the list proposed by Sarmento et al. compared to the AHRQ list and the AHRQ method compared to the Victorian list. Despite the results found, the choice of a list should always take into account the health context of the study population as well as the diseases included in the list, as these factors impede the replacement of one list by another. Nevertheless, it is important to state that we should compare comparable indicators and ACSC lists cannot be used interchangeably.

## Figures and Tables

**Table 1:** Inclusion and Exclusion Criteria of Each Ambulatory Care Sensitive Conditions List.

		AHRQ	CIHI	Victorian ACSC Study	Sarmento et al.
Diseases included	Diabetes	X (short- and long-term)	X	X	X
	Uncontrolled diabetes	X			
	Lower-extremity amputation among patients with diabetes	X			
	Chronic obstructive pulmonary disease	X	X	X	X (and chronic bronchitis)
	Hypertension	X	X	X	X
	Heart failure	X	X (and pulmonary edema)	X (congestive)	X
	Angina		X	X	
	Atrial fibrillation				X
	Dehydration	X		X (and gastroenteritis)	X (and hydroelectrolytic changes)
	Pneumonia	X (bacterial pneumonia)		X (and influenza)	X
	Urinary tract infection	X		X (pyelonephritis)	X
	Asthma	X (in young adults)	X	X	X
	Grand mal status and other epileptic convulsions		X	X (convulsions and epilepsy)	
	Other vaccine-preventable			X	
	Iron deficiency anemia			X	X
	Nutritional deficiencies			X	
	Perforated/Bleeding ulcer			X	
	Cellulitis			X	X (acute skin infections)
	Pelvic inflammatory disease			X	
	Ear, nose, and throat infections			X	
	Dental conditions			X	X
	Gangrene			X	
	Uterine cervical cancer				X
	Colorectal cancer				X
	Dementia				X
	Depression				X
	Gastroenteritis				X
	Obesity				X
	Thromboembolic venous disease				X
	Voluntary termination of pregnancy				X
Exclusion criteria	Age	Admissions younger than 18 years old	Admissions older than 75 years old	No age limit	Admissions younger than 18 years old
	Procedure codes	X	X	X	
	Obstetric admissions	X			
	Transfers from other institutions	X			
	Missing gender	X	X		
	Missing year	X			
	Missing age	X			
	Missing principal diagnosis	X			
	Missing residence	X			
	Records with discharge as death		X		
	Newborn, stillbirth, or cadaveric donor records		X		

Notes: ACSC: ambulatory care sensitive condition; AHRQ: Agency for Healthcare Research and Quality; CIHI: Canadian Institute for Health Information.

**Table 2:** Number of Hospitalizations Per Year and Respective Proportion of Avoidable Hospitalizations by Four Distinct Lists.

	Avoidable Hospitalizations, *n* (%)				
Year	AHRQ	CIHI	Victorian	Sarmento et al.	Total hospitalizations
2011	87,680 (9.8%)	20,386 (2.3%)	99,002 (11.1%)	118,685 (13.3%)	893,977
2012	91,040 (10.2%)	20,871 (2.3%)	102,349 (11.5%)	125,049 (14.0%)	890,484
2013	94,099 (10.6%)	20,352 (2.3%)	104,580 (11.8%)	126,195 (14.3%)	884,566
2014	93,704 (10.8%)	19,822 (2.3%)	104,477 (12.0%)	125,641 (14.5%)	867,876
2015	95,216 (11.0%)	19,680 (2.3%)	106,594 (12.3%)	127,938 (14.8%)	866,252
Total	461,739 (10.5%)	101,111 (2.3%)	517,002 (11.7%)	623,508 (14.2%)	4,403,155

Notes: AHRQ: Agency for Healthcare Research and Quality; CIHI: Canadian Institute for Health Information. Total hospitalizations were calculated considering our study exclusion criteria (the same for all lists, based on outpatient care setting, patient residence information, statistical validity).

**Table 3:** Age–Sex-Adjusted Rate Per District According to Four Different Methods in 2011 and 2015 (Per 100,000 Inhabitants).

	Hospitalizations Per 100,000 Inhabitants							
	2011				2015			
District	AHRQ	CIHI	Victorian	Sarmento et al.	AHRQ	CIHI	Victorian	Sarmento et al.
Aveiro	1204.6	211.0	1060.2	1625.2	1109.0	199.4	1011.0	1521.3
Beja	754.2	143.3	721.4	1087.8	1025.3 *	228.6 *	976.4 *	1375.8 *
Braga	1019.6	185.9	951.7	1451.3	1217.8 *	195.3	977.9	1617.9 *
Bragança	1199.0	262.8	1138.4	1632.3	1143.0	234.9 †	1109.2	1624.7
Castelo Branco	1387.5	421.9	1543.6	1875.8	1398.5	423.0	1474.0	1837.6
Coimbra	1435.2	310.8	1094.6	1867.2	1430.5	249.4 †	1146.1	1875.1
Évora	767.7	188.9	697.2	1035.6	769.8	174.2	821.8 *	1060.1
Faro	984.7	181.6	796.7	1244.5	1057.5	197.5	846.4	1343.3
Guarda	962.9	236.4	788.1	1391.8	1023.7	268.5 *	867.0 *	1518.1
Leiria	1569.4	289.4	1186.6	2055.3	1548.0	242.6 †	1224.1	1880.3
Lisboa	1194.2	253.0	1123.9	1572.2	1139.2	238.8	1149.1	1586.5
Portalegre	1151.6	360.2	1046.7	1368.6	1292.9 *	249.6 †	1048.7	1493.8
Porto	1100.5	212.9	1098.3	1475.5	1067.0	210.8	1097.6	1437.7
Santarém	1080.1	245.7	1132.8	1636.0	1369.1 *	257.4	1213.4	1768.1
Setúbal	977.4	187.0	899.7	1304.8	1038.2	172.6	1036.6 *	1388.4
Viana do Castelo	1005.6	211.4	951.7	1391.1	1081.2	186.0 †	1026.3	1478.3
Vila Real	1384.3	308.6	1228.8	1853.6	1645.8 *	315.2	1306.9	2124.4 *
Viseu	1095.5	236.9	956.4	1523.8	1157.0	213.9	1088.5 *	1669.4
Mainland Portugal	1126.3	247.1	1023.1	1521.8	1195.2	236.5	1078.9	1588.9

Notes: AHRQ: Agency for Healthcare Research and Quality; CIHI: Canadian Institute for Health Information; *: shows an increase of 10% or more in the district rate values between the years 2011 and 2015; †: shows a decrease of 10% or more in district rate values between the years 2011 and 2015

**Table 4:** The Correlation between Lists Assessed in Terms of Spearman’s Rho.

	CIHI vs. AHRQ		CIHI vs. Victorian	AHRQ vs. Victorian
Year	*n*	Spearman’s rho [95% CI]	Spearman’s rho [95% CI]	Spearman’s rho [95% CI]
2011	18	0.787 [0.498; 0.920]	0.779 [0.485; 0.950]	0.876 [0.607; 0.975]
2012	18	0.829 [0.523; 0.948]	0.777 [0.473; 0.923]	0.798 [0.509; 0.925]
2013	18	0.934 [0.788; 0.983]	0.938 [0.796; 0.985]	0.880 [0.694; 0.952]
2014	18	0.761 [0.386; 0.950]	0.730 [0.318; 0.954]	0.860 [0.632; 0.946]
2015	18	0.602 [0.135; 0.894]	0.645 [0.152; 0.919]	0.822 [0.492; 0.937]
Overall	90	0.806 *	0.786 *	0.861 *
	AHRQ vs. Sarmento et al.		CIHI vs. Sarmento et al.	Victorian vs. Sarmento et al.
Year	*n*	Spearman’s rho [95% CI]	Spearman’s rho [95% CI]	Spearman’s rho [95% CI]
2011	18	0.893 [0.667; 0.981]	0.736 [0.316; 0.956]	0.899 [0.715; 0.975]
2012	18	0.893 [0.635; 0.987]	0.818 [0.501; 0.952]	0.841 [0.607; 0.960]
2013	18	0.911 [0.697; 0.994]	0.880 [0.638; 0.960]	0.874 [0.672; 0.966]
2014	18	0.957 [0.828; 0.994]	0.816 [0.534; 0.935]	0.853 [0.623; 0.943]
2015	18	0.903 [0.659; 0.994]	0.676 [0.292; 0.863]	0.820 [0.528; 0.950]
Overall	90	0.921 *	0.796 *	0.868 *

Notes: AHRQ: Agency for Healthcare Research and Quality; CIHI: Canadian Institute for Health Information. 95% CI (confidence interval) was calculated based on 1000 bootstrap samples. * 95% CIs were not calculated as there is a dependency between years.

**Table 5:** Comparison of Different Pairs of Lists in Terms of Information-Based Measure of Disagreement (IBMD) and Intraclass Correlation Coefficient (ICC).

	CIHI vs. AHRQ			CIHI vs. Victorian	
Year	*n*	IBMD [95% CI]	ICC [95% CI]	IBMD [95% CI]	ICC [95% CI]
2011	18	0.833 [0.816; 0.844]	0.604 [−0.058; 0.852]	0.815 [0.799; 0.829]	0.661 [0.094; 0.873]
2012	18	0.837 [0.826; 0.846]	0.596 [−0.080; 0.849]	0.816 [0.802; 0.826]	0.634 [0.021; 0.863]
2013	18	0.845 [0.832; 0.854]	0.586 [−0.107; 0.845]	0.827 [0.816; 0.835]	0.683 [0.153; 0.882]
2014	18	0.845 [0.830; 0.855]	0.569 [−0.153; 0.839]	0.827 [0.812; 0.840]	0.677 [0.138; 0.879]
2015	18	0.849 [0.833; 0.860]	0.477 [−0.397; 0.805]	0.833 [0.818; 0.844]	0.660 [0.091; 0.873]
Overall	90	0.842 *	0.561 *	0.823 *	0.654 *
	AHRQ vs. Victorian			AHRQ vs. Sarmento et al.	
Year	*n*	IBMD [95% CI]	ICC [95% CI]	IBMD [95% CI]	ICC [95% CI]
2011	18	0.142 [0.100; 0.185]	0.904 [0.744; 0.964]	0.333 [0.310; 0.353]	0.968 [0.914; 0.988]
2012	18	0.148 [0.100; 0.197]	0.903 [0.740; 0.964]	0.336 [0.309; 0.352]	0.960 [0.893; 0.985]
2013	18	0.133 [0.087; 0.183]	0.918 [0.781; 0.969]	0.327 [0.299; 0.345]	0.963 [0.900; 0.986]
2014	18	0.131 [0.087; 0.184]	0.889 [0.702; 0.958]	0.330 [0.304; 0.352]	0.968 [0.915; 0.988]
2015	18	0.141 [0.103; 0.187]	0.864 [0.636; 0.949]	0.320 [0.293; 0.340]	0.968 [0.913; 0.988]
Overall	90	0.139 *	0.898 *	0.330 *	0.965 *
	CIHI vs. Sarmento et al.			Victorian vs. Sarmento et al.	
Year	*n*	IBMD [95% CI]	ICC [95% CI]	IBMD [95% CI]	ICC [95% CI]
2011	18	0.878 [0.864; 0.888]	0.492 [−0.358; 0.810]	0.406 [0.373; 0.436]	0.895 [0.719; 0.961]
2012	18	0.882 [0.873; 0.888]	0.482 [−0.385; 0.806]	0.421 [0.387; 0.452]	0.877 [0.672; 0.954]
2013	18	0.886 [0.876; 0.893]	0.508 [−0.315; 0.816]	0.407 [0.376; 0.436]	0.890 [0.707; 0.959]
2014	18	0.887 [0.877; 0.893]	0.529 [−0.260; 0.824]	0.405 [0.377; 0.434]	0.865 [0.638; 0.949]
2015	18	0.888 [0.878; 0.896]	0.453 [−0.463; 0.795]	0.396 [0.362; 0.426]	0.853 [0.606; 0.945]
Overall	90	0.884 *	0.489 *	0.407 *	0.878 *

Notes: AHRQ: Agency for Healthcare Research and Quality; CIHI: Canadian Institute for Health Information. ICC: Two-way mixed-effects model (average measures). * 95% CIs were not calculated as there is a dependency between years.
